# Ticks and tick‐borne pathogens in selected abattoirs and a slaughter slab in Kumasi, Ghana

**DOI:** 10.1002/vms3.70030

**Published:** 2024-09-17

**Authors:** Stacy Amoah, Nancy Martekai Unicorn, Emmanuella Tiwaa Kyeremateng, Genevieve Desewu, Patrick Kwasi Obuam, Richard Odoi‐Teye Malm, Emmanuel Osei‐Frempong, Francisca Adai Torto, Stephen Kwabena Accorlor, Kwadwo Boampong, Sandra Abankwa Kwarteng, Seth Offei Addo, John Asiedu Larbi

**Affiliations:** ^1^ Department of Theoretical and Applied Biology College of Science KNUST Kumasi Ghana; ^2^ School of Public Health Kwame Nkrumah University of Science and Technology Kumasi Ghana; ^3^ Parasitology Department Noguchi Memorial Institute for Medical Research University of Ghana Accra Ghana

**Keywords:** abattoir, cattle, Ixodidae, spotted fever group rickettsiae, zoonoses

## Abstract

**Background:**

Ticks are vectors of pathogens that affect the health of animals and humans. With the constant trade of livestock across borders, there is the risk of new tick species invasion accompanied by the spread of infectious tick‐borne pathogens.

**Aim:**

This study sought to determine the diversity of tick species within abattoirs and a slaughter slab as well as identify the pathogens carried by these ticks.

**Methods:**

The ticks were collected from slaughtered cattle, identified and screened for pathogens using PCR and sequencing.

**Results:**

A total of 371 ticks were collected from slaughtered cattle across the three sampling sites: Kumasi abattoir (288, 77.63%), Akwatia Line slaughter slab (52, 14.02%) and Suame abattoir (31, 8.35%). The predominant species was *Amblyomma variegatum* (85.44%) with *Rhipicephalus sanguineus* (s.l.) (0.27%) as the least occurring species. Total nucleic acid from the tick pools was screened for pathogens based on the nucleoprotein gene region in the S segment of the Crimean–Congo haemorrhagic fever virus (CCHFV) genome, the 295‐bp fragment of the transposase gene of the *Coxiella burnetii* IS1111a element, the 560 bp segment of the ssrRNA gene of *Babesia* and *Theileria*, the 345 bp fragment of the *Ehrlichia* genus 16SrRNA gene and the rOmpA gene (OmpA) of *Rickettsia*. From the 52 tick pools screened, 40 (76.92%) were found positive for pathogen DNA. The pathogens identified were *Rickettsia africae* (69.23%), *Rickettsia aeschlimannii* (7.69%), *C. burnetii* (5.77%), uncultured *Ehrlichia* sp. (5.77%), *Candidatus* Midichloria mitochondrii (3.85%) and CCHFV (3.85%). A significant association was observed among *A. variegatum*, *Hyalomma rufipes*, *Hyalomma truncatum* and occurring tick‐borne pathogens *R. africae*, *R. aeschlimannii* and uncultured *Ehrlichia* sp. (*p* < 0.001).

**Conclusion:**

The findings show the occurrence of zoonotic pathogens, suggesting an increased risk of infections among the abattoir workers. There is a need to adopt control measures within the abattoirs to prevent pathogen spread.

## Graphical abstract

•*Amblyomma variegatum* was the predominant tick species.

•Zoonotic pathogens *R. africae*, *R. aeschlimannii*, *C. burnetii* and CCHFV were identified.

•Abattoir workers are at risk of zoonotic infections.

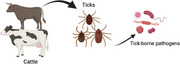

## 1 INTRODUCTION

Worldwide, about 80% of the cattle population, especially in tropical and subtropical countries, are significantly affected by tick infestation (Magesa et al., 2023). Tick infestation in cattle often results in direct consequences such as blood and weight loss, as well as indirect effects due to the role of ticks as vectors of infectious diseases (Sahara et al., 2019). Ticks are blood‐feeding ectoparasites that belong to the order Ixodida and are known vectors of various zoonotic diseases worldwide (Eisen et al., 2016). Nearly 10% of the 900 species of known ticks are capable of transmitting infectious pathogens to animals and humans, making ticks significant vectors of numerous diseases that affect animals and humans (Jongejan & Uilenberg, 2004).

It has been reported that the preferred ecological settings of each tick species influence where tick‐borne diseases are most likely to occur (Parola & Raoult, 2001). There are close to 50 indigenous tick species in Africa that are known to infect domestic and livestock animals. *Amblyomma, Hyalomma* and *Rhipicephalus* are three genera among these unique species that have the greatest impact on animal health (Reye et al., 2012). Humans get infected with tick‐borne pathogens either through tick bites or coming into contact with an infected animal (Telmadarraiy et al., 2015).

The geographic spread and infection rates of zoonotic pathogens spread by ticks have risen, and they are anticipated to constitute a significant risk to human health in the future (Estrada‐Peña & De La Fuente, 2014). In countries throughout Africa, the Middle East, the Balkans and Asia, the Crimean–Congo haemorrhagic fever virus (CCHFV), for example, causes severe outbreaks of viral haemorrhagic fever, characterized by a case fatality estimated to range from 10% to 40% (Shahhosseini et al., 2021). The zoonotic pathogen *Coxiella burnetii* is naturally present in more than 40 different tick species (Maurin & Raoult, 1999). Even though *C. burnetii* is transmitted through contact with infected animals or animal products, ticks can transmit infections as the bacteria are shed through their faeces (Angelakis & Raoult, 2010). Again, it has been observed that rickettsial infections have spread to non‐endemic areas due to increasing tick bites (Parola et al., 2013). In Africa, tick species *Amblyomma hebraeum* or *Amblyomma variegatum* are primarily responsible for the spread of *Rickettsia* species depending on the geographical location (Parola et al., 2005).

Limited studies conducted in Ghana indicate the occurrence of tick species and tick‐borne pathogens. Among these pathogens are *Rickettsia* spp., *C. burnetii* (Nimo‐Paintsil et al., 2022), CCHFV (Akuffo et al., 2016), *Anaplasma* spp. (Addo, Olivia, et al., 2023) and *Babesia* spp. (Bell‐Sakyi et al., 2004; Nagano et al., 2013), all of which have been identified in Ghana. These pathogens pose a threat to both animals and humans; hence, there is a need to prevent their spread. Abattoir employees face a heightened risk of zoonotic infections due to their direct contact with livestock. Unfortunately, many of these infections go unnoticed, unreported and misdiagnosed as a result of limited resources. Furthermore, animals can serve as reservoirs and amplifying hosts for zoonotic pathogens. This situation has been reported in the Upper East region of Ghana, where livestock from the abattoir were found to harbour zoonotic tick‐borne pathogens (Addo, Bentil, Yartey, et al., 2023). Given these factors, this study sought to identify tick species infesting slaughtered cattle in Kumasi and determine the presence of tick‐borne pathogens that could potentially infect the abattoir workers.

## 2 METHODS

**FIGURE 1:**
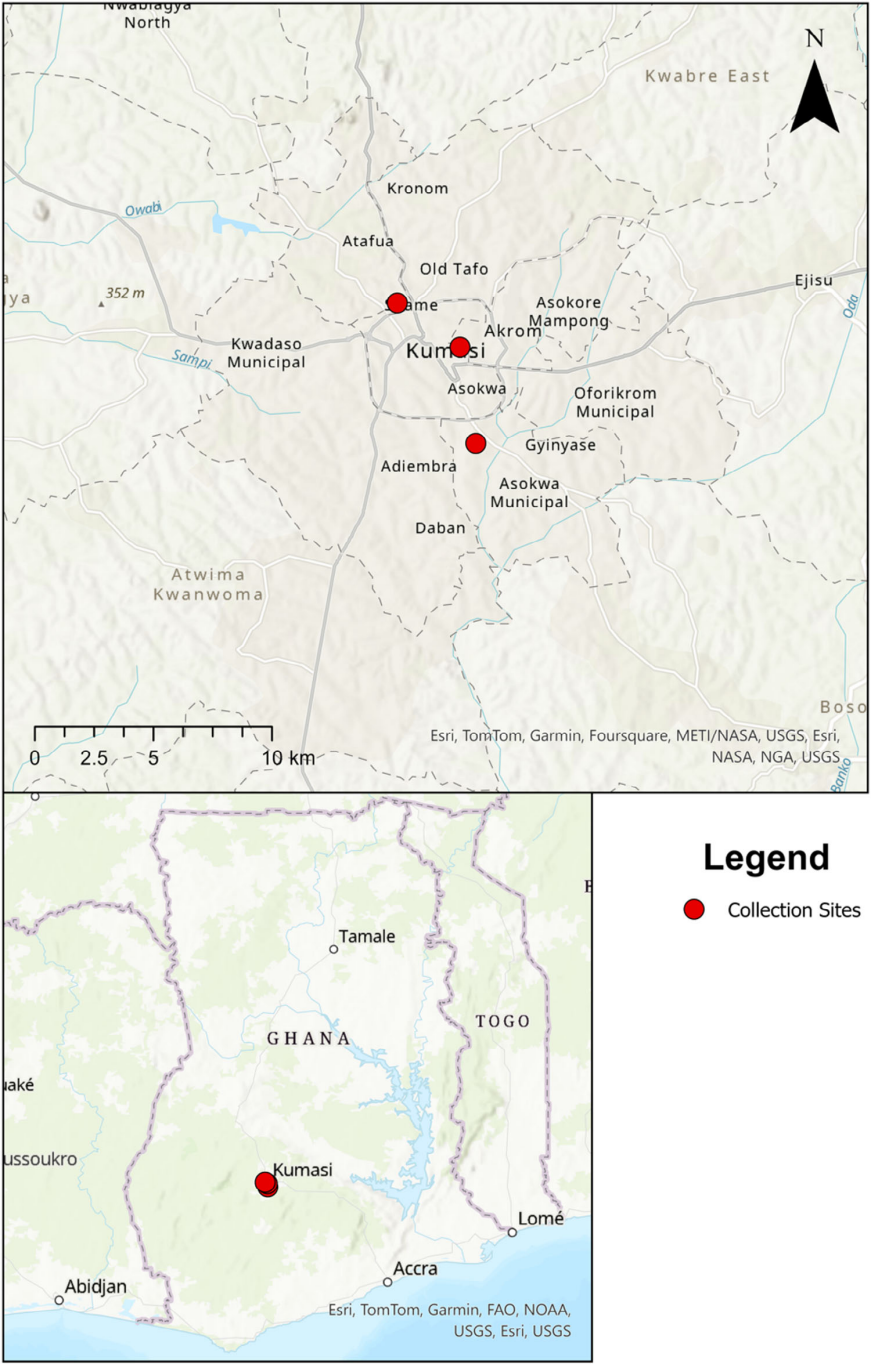
Map showing the tick collection sites.

The study was conducted in two different abattoirs and a slaughter slab within the Kumasi municipality of Ghana (Figure 1). The study sites were the Akwatia Line slaughter slab at Amakom, the Kumasi abattoir at Kaase and the Suame abattoir, where livestock from within the community and beyond are slaughtered daily. On average, the cattle slaughtered daily are about 200 in the Kumasi abattoir, 25 in the Akwatia Line slaughter slab and 30 in the Suame abattoir. After verbal consent, ticks were manually removed post‐slaughter from the cattle and placed in labelled 15 mL falcon tubes. The health status of the cattle could not be confirmed as they were slaughtered before the tick collections were done. The majority of the ticks were unfed; a few were partially engorged, and a lesser number were fully engorged. The ticks were sent to the laboratory where they were morphologically identified using taxonomic keys (Walker et al., 2003), pooled (1–10) per animal according to the specific species, sex and location and kept in 2 mL Eppendorf tubes containing RNAlater for storage at −80°C until molecular analysis.

## 3 TICK‐BORNE PATHOGEN DETECTION AND ANALYSIS

Total nucleic acid was extracted from each tick pool using the QIAamp Mini Kit following the manufacturer's instructions (Crowder et al., 2010). The presence of CCHFV was determined using an assay that amplifies the conserved region of the CCHFV genome, specifically the nucleoprotein gene region in the S segment (Atkinson et al., 2012). Furthermore, *C. burnetii* was detected using an assay that amplifies the 295‐bp fragment of the transposase gene of the *C. burnetii* IS1111a element (Klee et al., 2006). Samples with cycle threshold (Ct) values below 40 were deemed positive for CCHFV or *C. burnetii* after comparing their curves to those of the positive control. A 632 bp amplification was achieved using primers that target the rOmpA gene (OmpA) of *Rickettsia* to detect the presence of *Rickettsia* DNA in tick pools (Jiang et al., 2005). The occurrence of *Babesia*/*Theileria* DNA was detected using primers that target the 560 bp segment of the ssrRNA gene of *Babesia* and *Theileria* (Beck et al., 2009). Using primers that target the 345 bp fragment of the *Ehrlichia* genus 16SrRNA gene (Nazari et al., 2013), the presence of *Ehrlichia* and *Anaplasma* DNA was confirmed in the tick pools. The PCR products from the conventional PCRs were separated on 2% agarose gel, visualized using a Molecular Imager Gel Doc and positive products were purified and sequenced by Macrogen Europe B.V.

The pathogens from this investigation were subjected to sequence alignments and phylogenetic analysis using MEGA X (Kumar et al., 2018). The neighbour‐joining method was used to construct the phylogenetic tree. Thousand bootstrap replicates were used to calculate the confidence indices inside the phylogenetic trees, and the findings were shown on the branches as percentages. For the GenBank sequences used in the phylogenetic analysis, the different accession numbers and countries of origin have been mentioned.

### 3.1 Statistical analysis

The statistical analysis was carried out using STATA version 13, and the level of significance was set at *p* < 0.05. The chi‐square test was performed to assess the association between the occurrence of the detected pathogens and factors such as tick species and location.

We again assessed the pooled prevalence (minimum infection) of the different pathogens by location and species using proportions with exact confidence intervals. We then assessed the maximum likelihood estimation (MLE) using methods described in the PooledInfRate R package. All analysis was conducted in R version 4.3.3.

## 4 RESULTS

A total of 371 ticks were collected from cattle across the 3 sampling sites: Kumasi abattoir (288, 77.63%), Akwatia Line slaughter slab (52, 14.02%) and Suame abattoir (31, 8.35%). The ticks identified were of the genus *Amblyomma* (317), *Hyalomma* (40) and *Rhipicephalus* (14). The occurring tick species were *A. variegatum* (317, 85.44%), *Hyalomma truncatum* (21, 5.66%), *Hyalomma rufipes* (19, 5.12%), *Rhipicephalus* (*Boophilus*) sp. (13, 3.51%) and *Rhipicephalus sanguineus* (s.l.) (1, 0.27%).

## 5 PATHOGENS IDENTIFIED

From the 52 tick pools screened, 40 (76.92%) were found positive for pathogen DNA. Generally, the pathogens identified were *Rickettsia africae* (69.23%), *Rickettsia aeschlimannii* (7.69%), *C. burnetii* (5.77%), uncultured *Ehrlichia* sp. (5.77%), *Candidatus* Midichloria mitochondrii (3.85%) and CCHFV (3.85%). No *Babesia* or *Theileria* DNA was detected in the tick pools. The majority of pathogens were identified in tick pools from the Kumasi abattoir (61.54%) compared to the Akwatia Line slaughter slab (9.62%) and the Suame abattoir (5.76%). The MLE of *R. africae* in pools of *A. variegatum* was recorded as 24.43% (95% CI: 15.82, 39.55%) in the Kumasi abattoir, 13.80% (95% CI: 4.29, 34.92%) in Suame abattoir and 11.64% (95% CI: 3.22, 39.56%) in Akwatia Line (Table 1). In the Kumasi abattoir, the MLE of *C. burnetii* and CCHFV in pools of *A. variegatum* were 1.22% (95% CI: 0.32, 3.28%) and 0.81% (95% CI: 0.14, 2.64%), respectively (Table 1). A significant association was observed between *A. variegatum* and *R. africae* (*p* < 0.001), *H. rufipes* and *R. aeschlimannii* (*p* < 0.001), *H. truncatum* and uncultured *Ehrlichia* sp. (*p* < 0.001) as well as *H. rufipes* and Ca. M. mitochondrii (*p* < 0.001) (Table 2).

**TABLE 1:** Distribution of tick‐borne pathogens in the identified tick species.

Location	Species	Pathogens	Positives (n/N pools)	Positives (n/N ticks)	Pool infection rate (%)	Minimum infection rate (95% CI)	Maximum likelihood infection rate (95% CI)
Akwatia Line	*Amblyomma variegatum*	*Rickettsia africae*	3/4	3/38	75.0 (0.0–60.24)	7.89 (0.0–9.25)	11.64 (3.22–39.56)
	*Hyalomma rufipes*	*Rickettsia aeschlimannii*	1/1	1/2	100.0 (2.5–100.0)	50.0 (1.26–98.74)	–
	*Hyalomma truncatum*	*Rickettsia africae*	1/1	1/4	100.0 (0.0–97.5)	25.0 (0.0–60.24)	–
Kumasi abattoir	*Amblyomma variegatum*	CCHFV	2/30	2/253	6.67 (0.82–22.07)	0.79 (0.1–2.83)	0.81 (0.14–2.64)
	*Amblyomma variegatum*	*Coxiella burnetii*	3/30	3/253	10.00 (0.82–22.07)	1.19 (0.1–2.83)	1.22 (0.32–3.28)
	*Amblyomma variegatum*	*Rickettsia africae*	26/30	26/253	86.67 (0.82–22.07)	10.28 (0.1–2.83)	24.43 (15.82–39.55)
	*Hyalomma rufipes*	*Candidatus* Midichloria mitochondrii	2/3	2/17	66.67 (0.0–70.76)	11.76 (0.0–19.51)	11.64 (2.81–33.41)
	*Hyalomma rufipes*	*Rickettsia aeschlimannii*	3/3	3/17	100.00 (0.0–70.76)	17.65 (0.0–19.51)	–
	*Hyalomma truncatum*	*Rickettsia africae*	3/3	3/17	100.0 (0.0–70.76)	17.65 (0.0–19.51)	–
	*Hyalomma truncatum*	Uncultured *Ehrlichia* sp.	3/3	3/17	100.0 (0.0–70.76)	17.65 (0.0–19.51)	–
Suame abattoir	*Amblyomma variegatum*	*Rickettsia africae*	3/5	3/26	60.0 (14.66–94.73)	11.54 (2.45–30.15)	13.80 (4.29–34.92)

Abbreviation: CCHFV, Crimean–Congo haemorrhagic fever virus.

**TABLE 2:** Association among the identified pathogens, tick species and location.

		*Coxiella burnetii*		*Rickettsia africae*		*Rickettsia aeschlimannii*		Uncultured *Ehrlichia* sp.		*Candidatus* Midichloria mitochondrii		CCHFV	
	Total no. of pools	No. positive	*p*‐value	No. positive	*p*‐value	No. positive	*p*‐value	No. positive	*p*‐value	*No. positive*	*p*‐value	No. positive	*p*‐value
***Tick species***													
*Amblyomma variegatum*	39	3	0.9	32	<0.001	0	<0.001	0	<0.001	0	<0.001	2	0.952
*Hyalomma rufipes*	4	0		0		4		0		2		0	
*Hyalomma truncatum*	4	0		4		0		3		0		0	
*Rhipicephalus* (*Boophilus*) sp.	4	0		0		0		0		0		0	
*Rhipicephalus sanguineus*	1	0		0		0		0		0		0	
**Location**													
Akwatia Line	9	0	0.524	4	0.078	1	0.72	0	0.524	0	0.656	0	0.656
Kumasi abattoir	37	3		29		3		3		2		2	
Suame abbatoir	6	0		3		0		0		0		0	

Abbreviation: CCHFV, Crimean–Congo haemorrhagic fever virus.

*R. africae* in this study was 99%–100% similar to isolates from Benin (KT633262, KT633264), whereas *R. aeschlimannii* was 99% similar to an isolate from Spain (MW398876). Again, uncultured *Ehrlichia* sp. identified in this study was 99% similar to an isolate from Pakistan (MH250197), whereas the identified Ca. M. mitochondrii was 99% similar to a previous isolate from Ghana (OQ747945).

**FIGURE 2:**
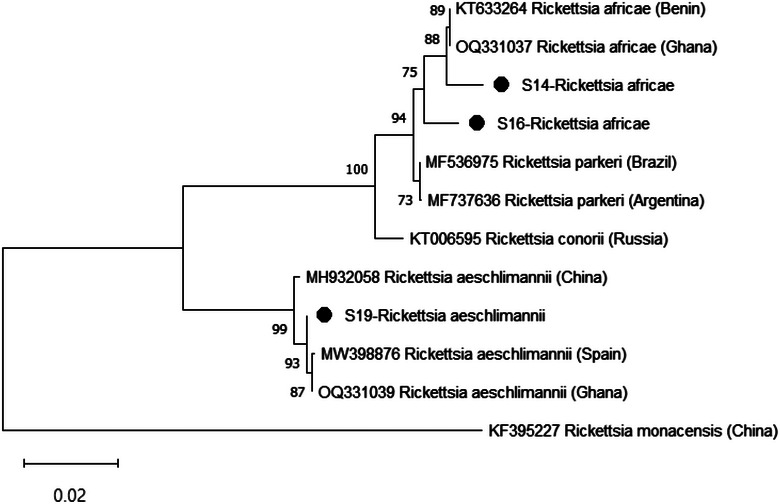
Phylogenetic tree of *Rickettsia* species based on the OmpA gene. The sequences from this study are indicated as S14, S16 and S19.

**FIGURE 3:**
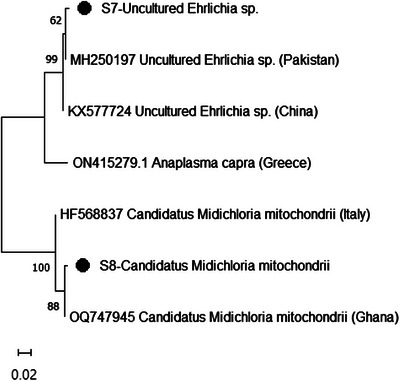
Phylogenetic tree of *Anaplasma* and *Ehrlichia* based on the 16SrRNA gene. The sequences obtained in this study are indicated as S7 and S8.

Based on the phylogenetic analysis, *R. africae* from this study clustered with isolates from Ghana (OQ331037) and Benin (KT633264) with bootstrap support of 75% (Figure 2). Furthermore, *R. aeschlimannii* from this study clustered with isolates from Ghana (OQ331039), Spain (MW398876) and China (MH932058) with bootstrap support of 99%. With a 99% bootstrap value, uncultured *Ehrlichia* sp. identified in this study clustered with isolates from Pakistan (MH250197) and China (KX577724) (Figure 3). Ca. M. mitochondrii from this study clustered with isolates from Ghana (OQ747945) and Italy (HF568837) with bootstrap support of 100%.

The sequences that were generated in this study have been deposited in GenBank: *R. africae* (OR248868 and OR248870), *R. aeschlimannii* (OR248865), uncultured *Ehrlichia* sp. (OR241132) and Ca. M. mitochondrii (OR241138).

## 6 DISCUSSION

In this study, ticks were collected from cattle across three sites in Kumasi. The majority of the ticks were collected from the Kumasi abattoir due to the large number of cattle slaughtered daily compared to the Akwatia Line slaughter slab or Suame abattoir. The predominant tick species in this study were *A. variegatum*, which has been reported as widely distributed in Ghana (Bell‐Sakyi et al., 1996; Walker & Koney, 1999) and occurs in more than 30 African countries (Walker et al., 2003). *A. variegatum* can cause severe damage to the livestock industry by reducing milk production, impairing animal growth and transmitting *Ehrlichia ruminantium* (Esemu et al., 2013; Stachurski, 2000). In Ghana, *C. burnetii*, *Rickettsia* species and CCHFV have been detected in *A. variegatum*, indicating its public health importance and a need for enforcing control measures (Addo, Bentil, Baako, et al., 2023; Akuffo et al., 2016; Nimo‐Paintsil et al., 2022). It should also be noted that other tick species identified in the study sites are capable of spreading various pathogens of veterinary and zoonotic importance (Reye et al., 2012). The *Hyalomma* species identified in this study can transmit diverse pathogens, including CCHFV in humans (Aktas et al., 2014; Gargili et al., 2017) and *Theileria annulata* in cattle (Mamman et al., 2020). *R. sanguineus* (s.l.) was the least tick species collected from the Kumai abattoir. This species is also known as the brown dog tick with a preference for dogs as hosts (Dantas‐Torres, 2008) but can be found infesting cattle at low numbers. Nonetheless, *R. sanguineus* (s.l.) can spread pathogens, such as *Ehrlichia*, *Babesia* and *Rickettsia* (Harrus et al., 1999; Tavassoli et al., 2013; Wikswo et al., 2007). Understanding the tick population dynamics and species prevalence is essential for developing effective tick control and management strategies, ultimately benefiting the health and well‐being of livestock and, consequently, the agricultural industry.

*R. africae* was detected mostly in *A. variegatum* and, to a lesser extent, *H. truncatum* in this study. *R. africae* is responsible for the disease African tick‐borne fever (Kelly et al., 1996) and is the principal cause of fever in travellers returning from Africa (Mediannikov, Diatta, et al., 2010). The findings of this study can be compared to previous studies in Ghana (Addo, Bentil, Baako, et al., 2023; Nimo‐Paintsil et al., 2022), Liberia and Guinea (Mediannikov et al., 2012) that found a high occurrence of *Rickettsia* DNA and *R. africae* in *A. variegatum*. There is an increased risk of *R. africae* transmission across the study sites, especially in light of its ability to be transmitted through transstadial and transovarial routes (Socolovschi et al., 2009) and the high occurrence of the principal vector *A. variegatum* (Parola et al., 2013). *R. aeschlimannii* was also identified in only *H. rufipes* in the Kumasi abattoir and Akwatia Line slaughter slab. This can be compared to studies in Ghana (Addo, Bentil, Baako, et al., 2023) and Nigeria (Kamani et al., 2015) that found *H. rufipes* infected with *R. aeschlimannii*. The findings show that different *Rickettsia* species occur in Ghana, and there is a need to fully determine the circulating species to create effective control strategies. *C. burnetii* was also identified in pools of *A. variegatum* in the Kumasi abattoir. This can be compared to previous studies in Ghana (Addo, Bentil, Baako, et al., 2023; Nimo‐Paintsil et al., 2022) and Senegal (Mediannikov, Fenollar, et al., 2010) that found ticks infected with *C. burnetii*. Reports from studies and the findings from this study suggest that ticks could play a role in the transmission of this pathogen.

CCHFV is an infectious pathogen transmitted primarily by species of the genera *Hyalomma*, although other tick species can transmit the virus (Spengler & Estrada‐Peña, 2018). In this study, *A. variegatum* from the Kumasi abattoir was found infected with the virus. This can be compared to a previous study in the same abattoir which found ticks, including *A. variegatum*, to be infected with CCHFV and pathogen exposure among the abattoir workers (Akuffo et al., 2016). The virus causes asymptomatic infections in wild and domestic animals (Bannazadeh & Aghazadeh, 2016; Fatemian et al., 2018) but leads to clinical symptoms such as haemorrhage and cardiovascular and neuropsychiatric abnormalities in humans (Whitehouse, 2004). Abattoir workers are at risk of infections; hence, there is a need to adopt effective preventive and control measures. A study in Ghana has reported the occurrence of *Ehrlichia canis* and *Ehrlichia minasensis* in ticks (Addo, Olivia, et al., 2023). In this study, uncultured *Ehrlichia* sp. was detected in *H. truncatum* from the Kumasi abattoir. This means that different *Ehrlichia* species are in circulation in Ghana. To develop an effective control measure, it will be necessary to conduct more surveillance activities to determine the species in circulation and their effects on livestock production. Ca. M. mitochondrii is a bacterial endosymbiont of ticks that is found inside the mitochondria and occurs frequently in *Ixodes ricinus* (Stavru et al., 2020). In this study, it was detected in *H. rufipes* in the Kumasi abattoir. It is not clear if this symbiont influences pathogen transmission, although a study reported a positive correlation between varied levels of Midichloria mitochondrii and the occurrence of pathogen *Rickettsia parkeri* (Budachetri et al., 2018). The possibility of simultaneous transmission of several infections to hosts, including humans and animals, is raised by the co‐occurrence of numerous pathogens in the same tick species. This study found coinfections of *R. africae* and CCHFV in *A. variegatum* as well as *R. africae* and uncultured *Ehrlichia* sp. in *H. truncatum*. Co‐feeding on the same host allows for the exchange of pathogens between tick species and the animal host, increasing the risk of pathogen transmission.

## 7 CONCLUSION

In this study, *A. variegatum* was the predominant tick species and was found infected with multiple zoonotic pathogen DNA, including *R. africae*, *C. burnetii* and CCHFV. The majority of these pathogens were identified in the Kumasi abattoir indicating an increased risk of infection to the abattoir workers in direct contact with the cattle. The findings highlight the necessity of tick management, surveillance and preventative efforts to safeguard human and animal populations from tick‐borne pathogens of public health and veterinary importance.

## AUTHOR CONTRIBUTIONS

**Stacy Amoah**; **Nancy Martekai Unicorn**; **Emmanuella Tiwaa Kyeremateng** and **Genevieve Desewu**: Data curation; investigation. **Patrick Kwasi Obuam**; **Richard Odoi‐Teye Malm**; **Emmanuel Osei‐Frempong**; **Francisca Adai Torto** and **Sandra Abankwa Kwarteng**: Investigation; writing – review and editing. **Stephen Kwabena Accorlor**: Data curation; formal analysis. **Kwadwo Boampong**: Data curation; writing – review and editing. **Seth Offei Addo**: Conceptualization; data curation; investigation; writing – original draft. **John Asiedu Larbi**: Conceptualization; formal analysis; project administration; supervision; writing – review and editing.

## CONFLICT OF INTEREST STATEMENT

The authors declare no conflicts of interest.

## FUNDING INFORMATION

None.

## ETHICS STATEMENT

None.

### PEER REVIEW

The peer review history for this article is available at https://publons.com/publon/10.1002/vms3.70030.

## Data Availability

All the data supporting this study are included in the article.
